# Hyperactive behavioral phenotypes and an altered brain monoaminergic state in male offspring mice with perinatal hypothyroidism

**DOI:** 10.1016/j.toxrep.2019.10.005

**Published:** 2019-10-07

**Authors:** Toyoshi Umezu, Taizo Kita, Masatoshi Morita

**Affiliations:** a Center for Health and Environmental Risk Research, National Institute for Environmental Studies, Tsukuba, Ibaraki 305-8506, Japan; b Graduate School of Food and Nutrition, Kyushu Nutrition Welfare University, Kitakyushu, Fukuoka 803-8511, Japan; c Graduate School of Agriculture, Ehime University, Matsuyama, Ehime 790-8577, Japan

**Keywords:** Thyroid hormone, Hyperactivity, Brain development, Monoaminergic neurotransmitter, Mice

## Abstract

Thyroid hormone (TH) is essential for normal brain development. TH insufficiency during early stages of development may increase the risk for attention deficit/hyperactivity disorder, in which malfunction of brain monoaminergic systems is likely involved. However, little is known about the effects of perinatal hypothyroidism on behaviors and brain monoaminergic systems in offspring mice. The present study examined in mice (1) whether perinatal hypothyroidism causes hyperactive behavioral phenotypes, (2) how perinatal hypothyroidism influences brain monoaminergic systems, and (3) whether hyperactive behavioral phenotypes are associated with the state of brain monoaminergic systems. When dams were exposed to propylthiouracil, offspring mice developed hypothyroidism during the perinatal period. Offspring mice with perinatal hypothyroidism exhibited hyperactive behavioral phenotypes such as hyper-ambulatory activity and an increased response rate in the two-way active avoidance test in a male-specific manner. Significant decreases in dopamine (DA) and serotonin turnover were observed in the striatum (ST), nucleus accumbens, hypothalamus, and hippocampus in male mice with perinatal hypothyroidism. A significant correlation between ambulatory activity and DA turnover in the ST and an augmented ambulatory response to the DA reuptake inhibitor bupropion suggested that DA in the ST was involved in the hyper-ambulatory activity in mice with perinatal hypothyroidism.

## Highlights

• Pregnant mice were exposed to PTU to induce perinatal hypothyroidism (HypoTH) in pups.
• Male offspring with HypoTH showed hyperactive behavioral phenotypes.
• Dopamine (DA) turnover decreased in the striatum of male offspring with HypoTH.
• Degree of DA turnover in the striatum correlated with degree of locomotion.
• Male offspring with HypoTH showed augmented locomotor response to bupropion.

## Graphical abstract

**Figure:**
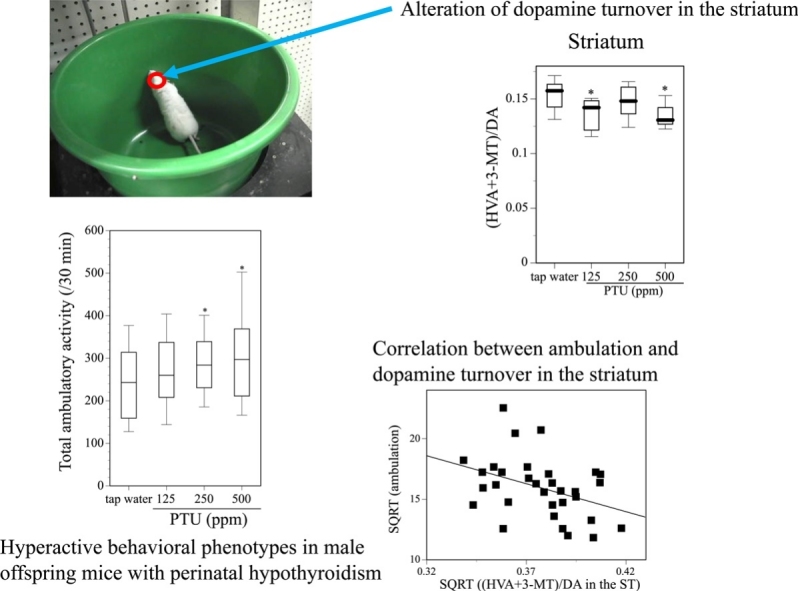

## 1 Introduction

Exposure to artificial chemicals may increase the risk of neurodevelopmental disorders [9]. Many artificial chemicals possess the potential to disrupt thyroid hormone (TH) [5,17], which is essential for normal brain development [4]. A high rate of attention deficit/hyperactivity disorder (ADHD), of which the major symptoms are hyperactivity, inattention, and impulsiveness, is observed in children with iodine deficiency, which causes TH insufficiency [24,39] and in children with resistance to TH [15] that is caused by mutation of a TH receptor beta isoform (TRβ) [32]. These observations suggest that TH disruption during early life stages impacts brain development and increases the risk of ADHD.

Risk factors for ADHD in humans produce ADHD-like behavioral phenotypes in mice, suggesting that mice are a useful animal species to test the potential of putative factors that induce ADHD-like neurodevelopmental disorders. Manipulation of the candidate gene for ADHD identified in humans [11,14,22,41] produces ADHD-like behavioral phenotypes in mice [21]. Resistance to TH increases the risk of ADHD [15]. Similarly, mice expressing human mutant TRβ1 exhibit ADHD-like behavioral phenotypes [33]. Hypothyroidism during early life stages may increase the risk of ADHD [24,39]. It is observed that TH insufficiency produces attention-deficit and hyperactive behavioral phenotypes in rats [25]. However, behavioral effects of hypothyroidism during development in mice have not been elucidated well.

Malfunction of brain monoaminergic systems may be involved in the etiology of ADHD [23]. Human genetic studies indicate that the dopamine (DA) transporter, DA receptor 1, and serotonin (5-hydroxytryptamine; 5-HT) receptor 1B are relevant to ADHD [11,14,22,41]. Mice lacking these genes exhibit ADHD-like behavioral phenotypes, showing that DA and 5-HT are involved in ADHD-like behavioral phenotypes [6,42,44]. TRα- and TRβ-knock out (KO) mouse studies indicate that normal development of brain monoaminergic systems requires both TRα and TRβ [27]. TRα and TRβ play different roles in brain monoaminergic systems. How hypothyroidism influences development of brain monoaminergic systems in mice remains unclear.

In the present study, in mice, we focused on (1) whether perinatal hypothyroidism causes hyperactive behavioral phenotypes, (2) how perinatal hypothyroidism influences brain monoaminergic systems, and (3) whether behavioral phenotypes are associated with the state of brain monoaminergic systems. The TH synthesis inhibitor propylthiouracil (PTU) was used to induce hypothyroidism. In mice, midbrain DA neurons start to extend axons at GD11.5, and 5-HT neurons start extending axons at approximately GD11, followed by increase in axonal projection density until PND60 [26]. Although no studies have described the expression of TH receptors in the fetal mouse brain, studies in rats have shown that TH receptors start to express on GD14, the expression rapidly increase after birth and become a peak at PND6 [2,29]. Considering these findings, mice were exposed to PTU from gestational day (GD) 15 to postnatal day (PND) 25 in the present study.

## 2 Materials and methods

### 2.1 Animals

ICR (or CD-1) strain mice (parent animals were purchased from Clea Japan, Tokyo, Japan) were used. Mice were housed in aluminum cages with stainless-steel mesh tops and paper bedding. Commercial solid food (Clea Japan) and tap water were provided *ad libitum*. The cages were placed in a room artificially illuminated by fluorescent lamps on a 12L:12D schedule (light period: 07:00-19:00) at a room temperature of 25 ± 1 °C.

Total number of animals used for each treatment group were; tap water group; dams; 22, male pups; 134, female pups; 70, 125 ppm PTU group; dams; 20, male pups; 110, female pups; 53, 250 ppm PTU group; dams; 22, male pups; 115, female pups; 55, 500 ppm PTU group; dams; 20, male pups; 104, female pups; 51.

All experiments were performed following approval by the Committee for Experimental Animals of the National Institute for Environmental Studies, Japan.

### 2.2 Drugs

Propylthiouracil (6-Propyl-2-thiouracil or 2,3-Dihydro-6-propyl-2-thioxo-4(1 H)-pyrimidinone; PTU) and bupropion HCl (BUP) were purchased from Sigma-Aldrich (Tokyo, Japan). PTU was dissolved in tap water at concentrations of 125, 250, or 500 ppm, and the solution was available *ad libitum* as drinking water to pregnant females. BUP was prepared in 0.9% NaCl (Nacalai Tesque, Kyoto, Japan) solution (saline) and given to offspring mice. The administration volume was 1 mL/100 g body weight regardless of the dosage.

### 2.3 Serum T4 assay

Serum T4 levels in collected blood samples from dams and male and female offspring mice were measured by radioimmunoassay using DPC・total T4 kits (Yatoron Co., Ltd. (present: LSI Medience Co., Ltd.), Tokyo, Japan) according to the company’s instructions. The limit of detection was 1.0 μg/dL.

### 2.4 Ambulatory activity

Ambulatory activity of the mice was measured using a tilt-type ambulometer (SAM-10; O’Hara and Co., Tokyo, Japan). The apparatus is explained in detail elsewhere [37].

### 2.5 Two-way active avoidance test

#### 2.5.1 Apparatus

Two-way active avoidance test was conducted using the apparatus previously reported [19,35]. Briefly, two identical experimental chambers (AA-3202A, O’Hara & Co.) was individually placed in a soundproof box (AO-4210, O’Hara & Co.). Movement of mice in the experimental chamber was detected using two sets of infrared photo beam sensors placed horizontally in the chamber. The floor consisted of a stainless-steel grid wired to pass an electric current. A speaker and lamp for presenting the warning stimulus (800-Hz tone and light) was set in the center of the ceiling of the chamber. The experimental chambers were connected to a personal computer *via* an interface (AA-1020 T, O’Hara & Co.), and computer software (O’Hara & Co.) was used to control the avoidance schedule and record data.

#### 2.5.2 Two-way active avoidance schedule

Experimental conditions of two-way active avoidance test was same to those in the previous studies [19,35]. Briefly, the temporal parameters were an inter-trial interval of 20 s and a warning (conditioned) stimulus (tone signal and light) duration of 5 s, followed by a maximum of 5 s of electric shock (unconditioned stimulus) with a conditioned stimulus if the mouse did not make an avoidance response. The shock (unconditioned stimulus) was a 50-Hz AC current (50 V, 0.2 mA) and delivered to the animal’s foot through the floor grid of the chamber. The response rate (number of photo beam interruptions/min) and avoidance rate (number of avoidance responses/number of avoidance trials) during each session were the indices for the avoidance response.

### 2.6 Assay for brain monoamine contents

Mice were sacrificed by decapitation, and the brain was removed and placed on ice. The frontal cortex, hypothalamus (HYPO), striatum (ST), hippocampi (HIPP), and nucleus accumbens (NAcb) were bilaterally dissected out, frozen in liquid nitrogen, and stored at −135 °C. Tissue was sonicated in 0.4 N perchloric acid and centrifuged at 17,760 × *g* for 15 min at 4 °C. The supernatant was frozen in liquid nitrogen and stored at −135 °C until the assay. DA, 3,4-dihydroxyphenylacetic acid (DOPAC), homovanillic acid (HVA), 3-methoxytyramine (3-MT), 5-HT, and 5-hydroxyindoleacetic acid (5-HIAA) levels were measured using high performance liquid chromatography with electrochemical detection (HPLC/ECD). The mobile phase (0.1 M citric acid, 15% MeOH, 0.1 mM octane sulfonic acid, and 0.1 mM Na_2_EDTA adjusted to pH 2.5) was added at 1.0 mL/min by the HPLC/ECD system (L-5000; Yanaco, Kyoto, Japan) to a 4.6 mm × 250 mm ODS-C18 column (Nacalai, Kyoto, Japan) maintained at 19 °C. Monoamines were detected with an electrochemical detector (VMD-101A; Yanaco) using a glassy carbon electrode maintained at +750 mV relative to an Ag/AgCl reference electrode. Protein concentration was determined using a BCA protein assay kit (Pierce, Rockford, IL, USA).

### 2.7 Experimental procedures

#### 2.7.1 Perinatal exposure to PTU, measurement of growth, and observation of eye opening in offspring mice

The day when a plug was observed in the dam was defined as GD0. Each dam was individually kept in a cage. Administration of 125, 250, or 500 ppm PTU solution was started on GD15. Tap water was given to control dams. The dams were observed every day, and the day when delivery was observed was defined as PND0. On PND0, the litter size and body weight of offspring were measured. Every 3–4 days after delivery, the body weight and eye opening of offspring were checked. According to our preliminary study, PND25 was set as the day for weaning in this study, as apparent growth retardation was observed in PTU-exposed offspring. On PND25, male and female offspring were separated, and their body weight was measured. Exposure to PTU was terminated on PND25, and tap water was given to all offspring mice thereafter. After weaning, the body weight of offspring was measured every 3–4 days.

#### 2.7.2 Blood collection for the serum T4 assay

On PND25, dams and one or two male and female offspring randomly selected from each litter were deeply anesthetized, and blood was transcardially collected. The serum samples of the collected blood were stored and subjected to T4 measurement at a later time.

#### 2.7.3 Behavioral examination

##### 2.7.3.1 Ambulatory activity

Ambulatory activity of male and female offspring mice was measured 2–4 months after birth. Mice were individually placed in the activity cages of the ambulometer, and the ambulatory activity was continuously measured for 30 min.

In the test to evaluate the response to the ambulation-promoting effect of BUP, individual mice were placed in the activity cages, and 30 min later, saline or 1.25, 2.5, 5, or 10 mg/kg BUP was subcutaneously administered, followed by measurement of ambulatory activity for 60 min. The test was repeated in the same mice starting with saline, followed by BUP challenges from a low dose to a high dose at 3- to 4-day intervals.

##### 2.7.3.2 Two-way active avoidance test

The two-way active avoidance test was performed 6–8 months after birth in male offspring mice and 8–10 months after birth in female offspring mice. Mice were individually placed in the experimental chambers and underwent an avoidance session for 40 min (80 trials) in 1 day. The avoidance session was repeated in the same mice for 5 consecutive days.

#### 2.7.4 Brain monoamine content assay

The brain monoamine content assay was conducted on male offspring mice. Ambulatory activity was measured on PND46-48. Mice were individually placed in the activity cages of the ambulometer, and the ambulatory activity was continuously measured for 30 min. After measurement of the ambulatory activity, the mice were returned to their home cages. The mice were sacrificed by decapitation on PND60, and the brains were collected. The samples were kept at −135 °C and later subjected to the monoamine content assay using the HPLC/ECD system.

### 2.8 Statistical analyses

P < 0.05 was considered statistically significant in all statistical analyses.

Changes in body weight were analyzed using repeated measures analysis of variance (ANOVA), followed by one-way ANOVA for each PND. The difference between tap water-treated controls and each PTU-exposed experimental group was tested using Dunnett’s test. The day of eye opening was analyzed using the Wilcoxon test.

As data were not distributed normally, ambulatory activity was analyzed using the Kruskal-Wallis or Wilcoxon tests. Similarly, monoaminergic neurotransmitter contents, metabolite contents, and turnover in the five brain regions were also analyzed using the Wilcoxon test. Relationships between ambulatory activity and each monoaminergic neurotransmitter content, metabolite content, and turnover in the five brain regions were examined with regression analysis after square root transformation of the ambulatory activity and the monoaminergic indices. The best-fit regression equation was obtained with the least-squares method. The significance of the best-fit regression equation was evaluated with ANOVA. Correlations between the ambulatory activity and each monoaminergic index were also evaluated using the coefficient of determination (R^2^).

Response rate and avoidance rate in the two-way active avoidance test were analyzed using two-way ANOVA, followed by one-way ANOVA and Dunnett’s test.

## 3 Results

### 3.1 Serum T4 and physical growth

**Fig. 1:**
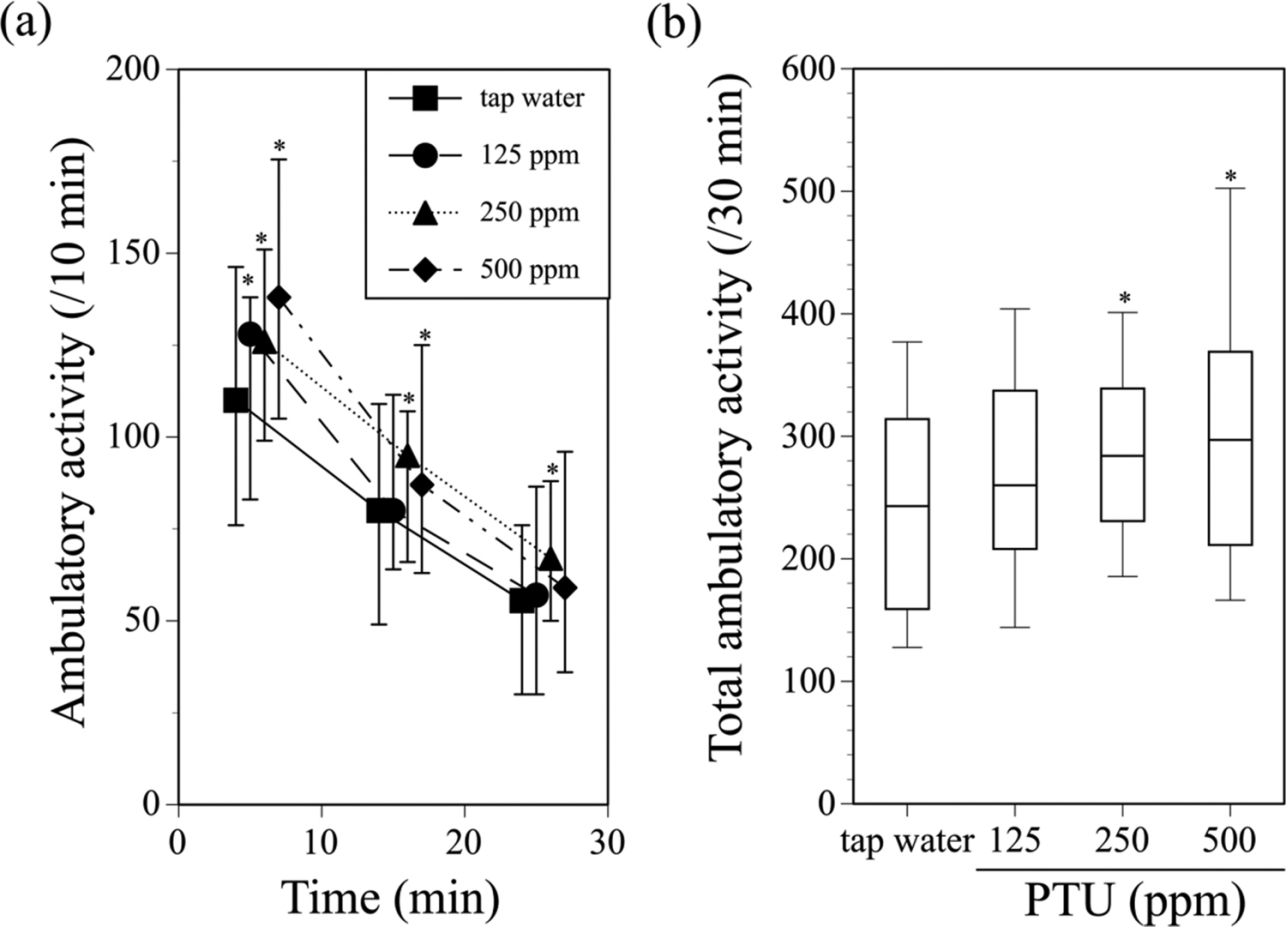
Ambulatory activity of male offspring mice exposed to 125, 250, or 500 ppm PTU from GD15 to PND25 through their dams. Tap water was administered to the dams of the control male offspring during the same period. Ambulatory activity was measured 2–4 months after birth. (a) Alterations in ambulatory activity during the 30-min period after introduction of mice into the bucket-like activity cages of the ambulometer. Symbols show median values of ambulatory activity for each 10-min period plotted against the midpoint of the measurement period, and vertical lines denote the first and third quartiles. (b) Total ambulatory activity for 30 min after introduction of the mice into the activity cages. Data are shown using a box plot, in which thick black lines indicate median, boxes indicate the 1st and 3rd quartiles and vertical lines indicate maximum and minimum values. *P < 0.05 compared with the tap water-administered control, assessed with the Wilcoxon test. N = 79–105 animals per PTU concentration.

PTU solution at a concentration of 125, 250, or 500 ppm was administered to dams from GD15 to PND25. Tap water was given to control dams. On PND25, T4 levels in serum of the dams and their male and female offspring were measured. In the control group, serum T4 levels were detected in all dams and all their male and female offspring (Data in Brief Article Fig. 1). Serum T4 levels were measurable in one dam, but were below the detectable level (1.0 μg/dL) in other dams in the 125 ppm PTU-administered group. Serum T4 was not detected in any male or female offspring of the 125 ppm PTU group. Serum T4 was not detected in any dams or male or female offspring in the 250 or 500 ppm PTU-administered groups (Data in Brief Article Fig. 1).

**Fig. 2:**
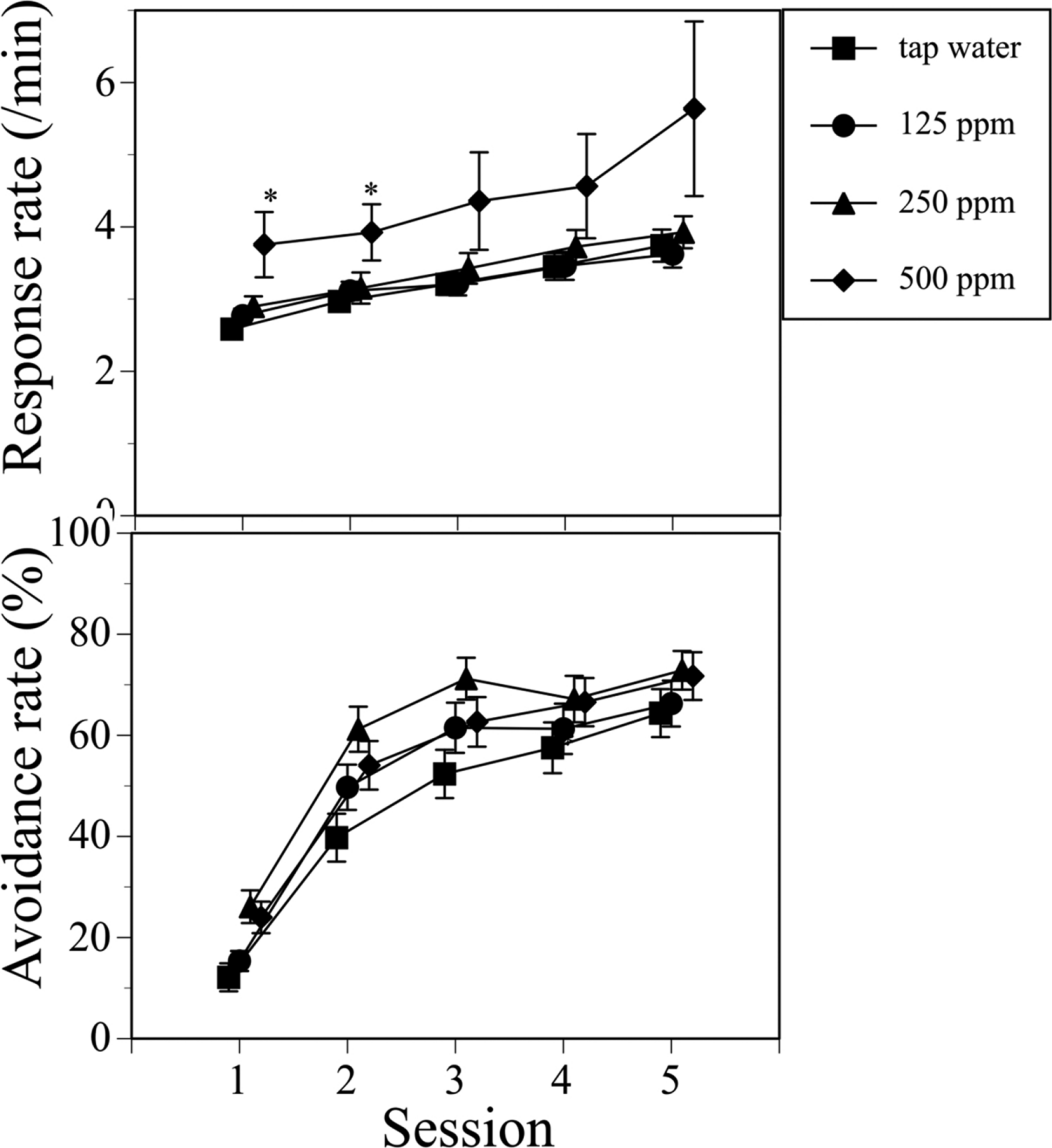
Alterations in the response rates (number of photo beam interruptions/min) and avoidance rates (number of avoidance responses/number of avoidance trials) in the two-way active avoidance test in male offspring mice in five sessions of the avoidance test. One avoidance test session consisted of 80 trials during a 40-min experimental period. Test sessions were conducted daily for 5 consecutive days. The avoidance test was performed 6–8 months after birth. Tap water and 125, 250, or 500 ppm PTU solution was administered to the dams of the offspring mice from GD15 to PND25. Upper panel: mean response rates are indicated by symbols; standard errors of the mean (SEM) are indicated by vertical lines. Lower panel: mean avoidance rates are indicated by symbols; SEM are indicated by vertical lines. Data were analyzed using two-way ANOVA, followed by one-way ANOVA and then Dunnett’s test. *P < 0.05 *vs*. tap water. N = 31–36 animals per group.

A delay in body weight gain is a typical physical sign of hypothyroidism during development. Perinatal PTU exposure retarded body weight gain in male and female offspring after birth in a concentration-dependent manner (Repeated measures ANOVA: male offspring; PTU dose F(3, 9) = 9.0736, P = 0.0044; Postnatal day F(26, 234) = 2063.7214, P < 0.0001; Interaction F(78, 234) = 3.4485, P < 0.0001; female offspring; PTU dose F(3, 10) = 3.0844, P = 0.0769; Postnatal day F(26, 260) = 1655.5591, P < 0.0001; Interaction F(78, 260) = 2.2202, P < 0.0001) (Data in Brief Article Fig. 2(a), (b)). Eye opening is a milestone in the development of pups. The day of eye opening was significantly delayed in PTU-exposed offspring in a concentration-dependent manner (Data in Brief Article Fig. 2(c)).

### 3.2 Effects on ambulatory activity

Fig. 1(a) shows the time course of ambulatory activity after introduction into the ambulometer in male offspring mice perinatally exposed to 125, 250, or 500 ppm PTU through their dams. Tap water was administered to the dams of the control offspring mice. The ambulatory activity of all groups was higher during the first 10-min period, followed by a gradual decrease in activity, showing adaptation of the mice to the experimental environment (Fig. 1(a)). Male offspring exposed to PTU showed significantly higher ambulatory activity. Total ambulatory activity of PTU-exposed animals for 30 min was also significantly higher than that of the animals given tap water (Fig. 1(b)). On the other hand, no effect of PTU exposure was observed on the ambulatory activity in female offspring (data not shown). Thus, perinatal exposure to PTU produced hyper-ambulatory activity in offspring mice in a male-specific manner.

### 3.3 Effects in the two-way active avoidance test

The response rate in the two-way active avoidance test is the average number of interruptions (during 40 min) of two sets of photo beam sensors horizontally placed in the experimental chamber. Thus, the response rate represents the degree of horizontal movement of the mouse in the experimental chamber.

Male offspring mice that were perinatally exposed to 500 ppm PTU exhibited a significantly increased response rate (Two-way ANOVA: PTU concentration, F(3, 130) = 3.2559, P = 0.0238; Session, F(4. 127) = 12.3038, P < 0.0001; Interaction F(12, 520) = 0.7636, P = 0.6883: One-way ANOVA; 1 st session: F(3, 130) = 4.6606, P = 0.004; 2nd session: F(3, 130) = 3.4531, P = 0.0185; 3rd session: F(3, 130) = 2.337, P = 0.0767; 4th session: F(3, 130) = 1.8067, P = 0.1491; 5th session: F(3, 130) = 2.376, P = 0.073) (Fig. 2). On the other hand, no effect of PTU exposure was observed on the response rate in female offspring (data not shown).

A change in the avoidance rate according to the number of trials and/or sessions indicates that mice have learned the avoidance response. No significant effect of PTU exposure was observed on the acquisition process in the male mice (Two-way ANOVA: PTU dose, F(3, 130) = 2.6247, P = 0.0533; Session, F(4, 127) = 150.2645, P < 0.0001; Interaction, F(12, 520) = 1.2102, P = 0.2723). Thus, perinatal exposure to PTU produced enhanced horizontal motor activity in the avoidance test in offspring mice in a male-specific manner.

### 3.4 Effects on the brain monoaminergic state and correlation between the brain monoaminergic state and ambulatory activity

Different offspring mice given tap water or 125–500 ppm PTU solution from GD15 to PND25 were prepared, and were used in the following experiment.

**Fig. 3:**
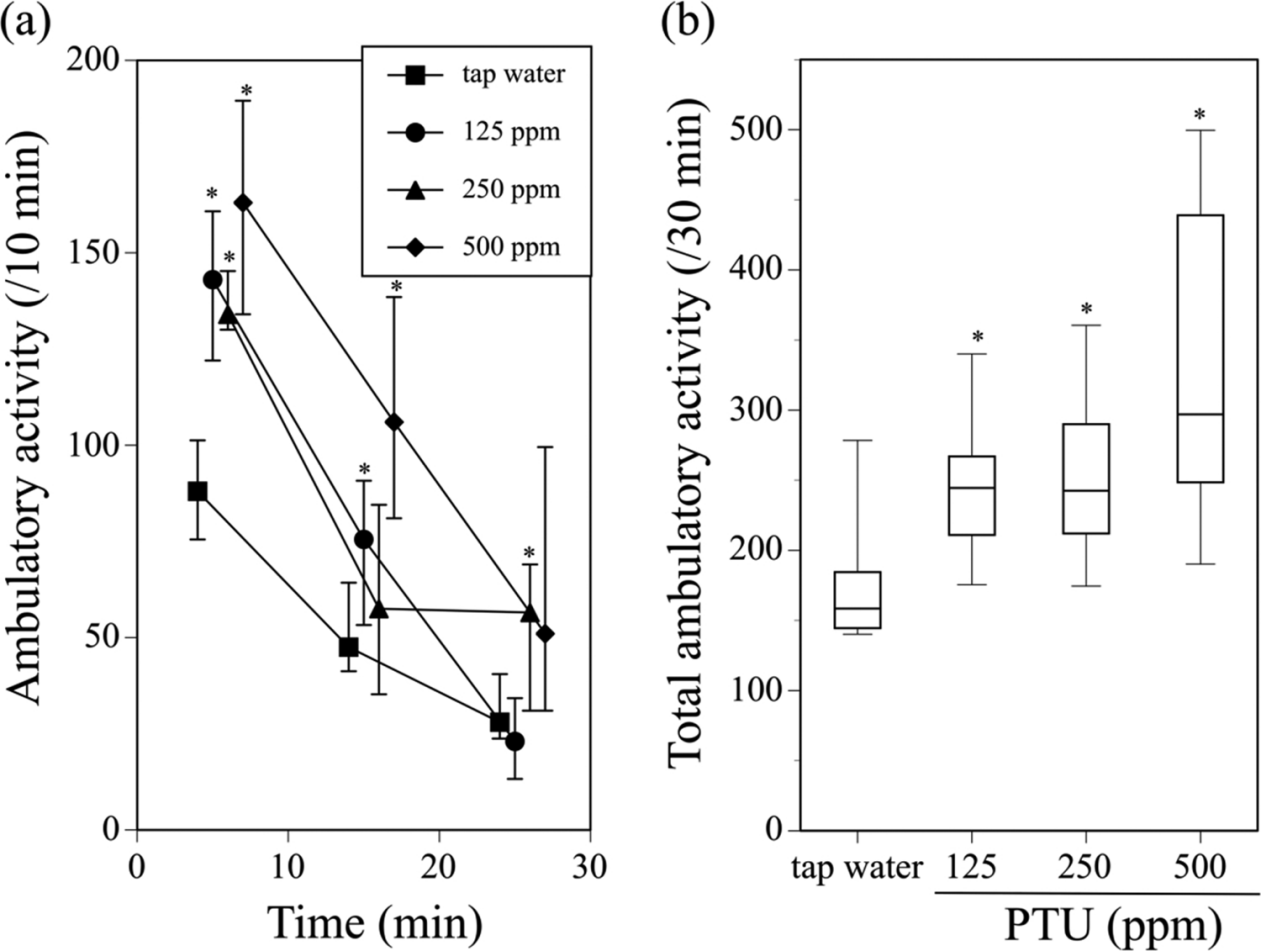
Ambulatory activity of male offspring mice exposed to 125, 250, or 500 ppm PTU from GD15 to PND25 through their dams. Tap water was administered to the dams of the control male offspring during the same period. Ambulatory activity was measured on PND46-48. (a) Alterations in ambulatory activity during the 30-min period after introduction of mice into the bucket-like activity cages of the ambulometer. Symbols show median values of ambulatory activity for each 10-min period plotted against the midpoint of the measurement period, and vertical lines denote the first and third quartiles. (b) Total ambulatory activity for 30 min after introduction of the mice into the activity cages. Data are shown using a box plot. *P < 0.05 compared with the tap water-administered control, assessed with the Wilcoxon test. N = 8 animals per group. The mice were sacrificed by decapitation, and the brains were collected on PND60. The monoaminergic state in five brain regions of these mice was examined, and the results are shown in Fig. 4 and Supplementary Figs. 2–4 are presented in Data in Brief.

The ambulatory activity was measured in the male offspring mice. Male offspring that were exposed to PTU showed significantly enhanced ambulatory activity (Fig. 3(a)). The total ambulatory activity of the PTU-exposed animals for 30 min was also significantly higher than that of animals given tap water (Fig. 3(b)).

**Fig. 4:**
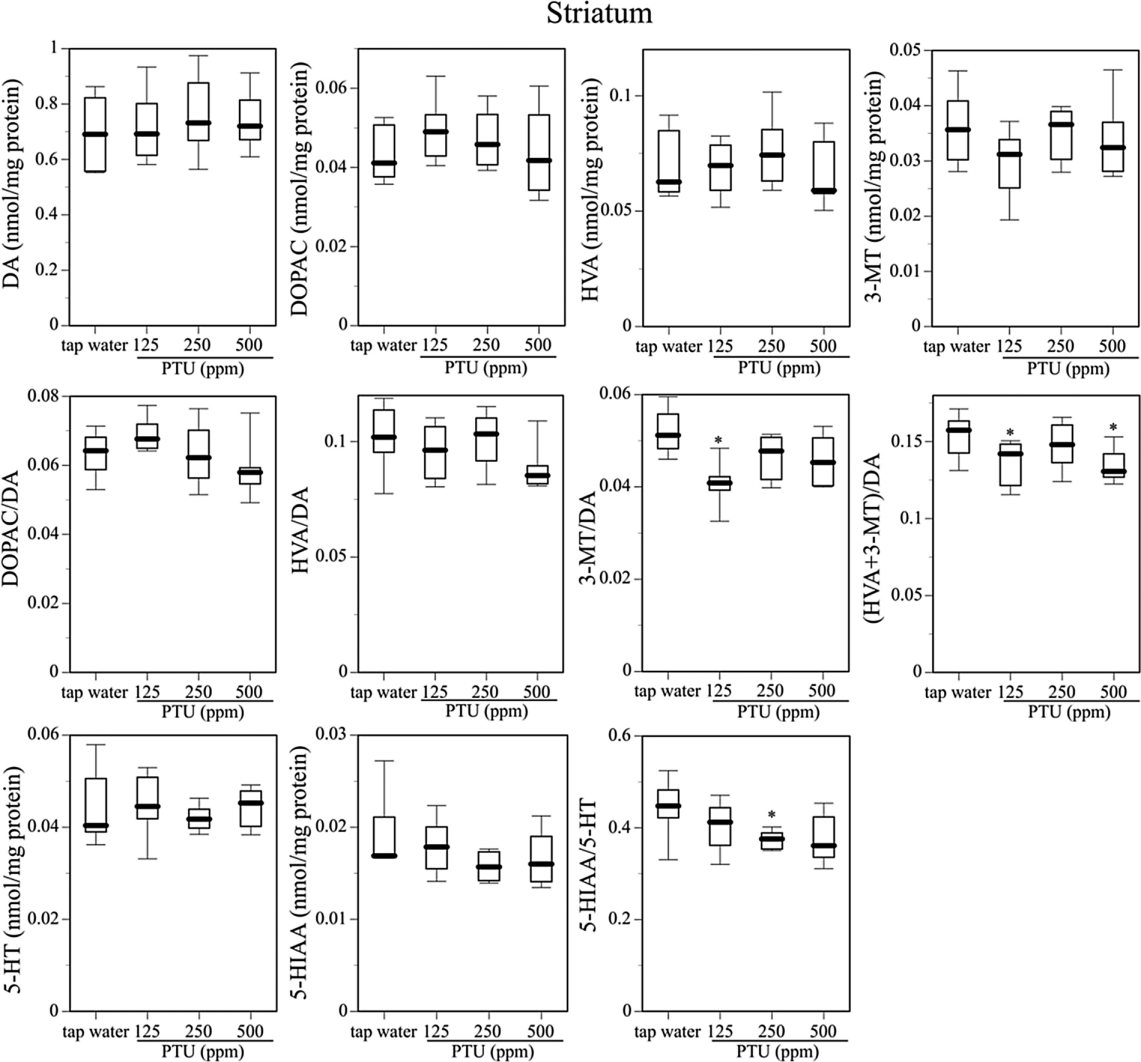
Contents of DA and DA metabolites (DOPAC, HVA, 3-MT), contents of 5-HT and the 5-HT metabolite (5-HIAA), DA turnover (DOPAC/DA, HVA/DA, 3-MT/DA, (HVA+3-MT)/DA), and 5-HT turnover (5-HIAA/5-HT) in the striatum (ST) of the mice given tap water or 125, 250, or 500 ppm PTU solution from GD15 to PND25. Data are shown using a box plot. *P < 0.05 compared with the tap water-administered control, assessed with the Wilcoxon test. N = 8 animals per group.

The brain monoaminergic state in these male offspring mice differed significantly. Fig. 4 shows the contents of DA; the DA metabolites DOPAC, HVA, and 3-MT; 5-HT; and the 5-HT metabolite 5-HIAA in the ST of the mice. The DA turnover and 5-HT turnover in the ST are also presented in Fig. 4. Perinatal exposure to 125–500 ppm PTU did not influence the contents of DA, DA metabolites, 5-HT, or the 5-HT metabolite, but did influence the DA turnover and 5-HT turnover. 3-MT/DA was significantly decreased in the 125 ppm PTU-exposed offspring, and (HVA+3-MT)/DA was significantly decreased in the 125 and 500 ppm PTU-exposed offspring. In addition, 5-HIAA/5-HT was significantly decreased in the 250 ppm PTU-exposed offspring.

**Fig. 5:**
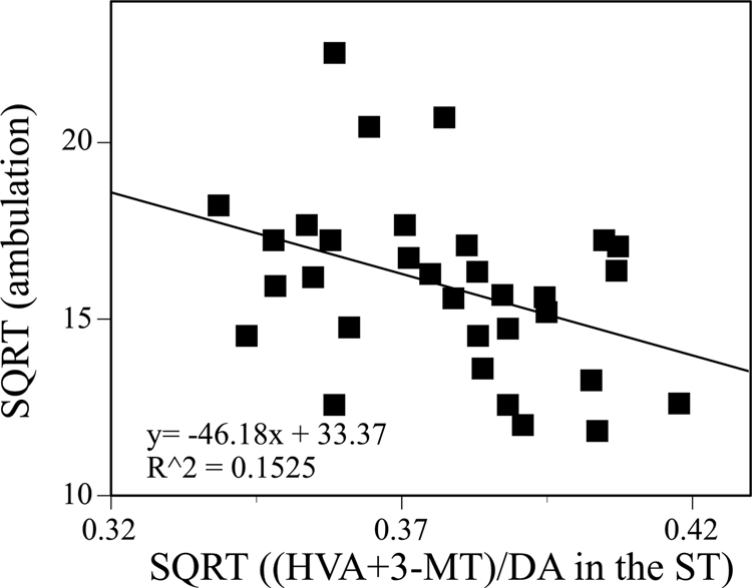
Correlation between the ambulatory activity and (HVA+3-MT)/DA in the striatum (ST) of the mice given tap water or 125, 250, or 500 ppm PTU solution from GD15 to PND25. SQRT indicates the square root transformation of the measurements. Data were analyzed using regression analysis, and the best fit regression equation and coefficient of determination (R^2^) are also shown in this figure.

Perinatal exposure to PTU influenced the content of 3-MT, 3-MT/DA, (HVA+3-MT)/DA, and 5-HIAA/5-HT in the NAcb (Data in Brief Article Fig. 3), the DOPAC content, HVA/DA, and 5-HIAA/5-HT in the HYPO (Data in Brief Article Fig. 4), and the 5-HT content and 5-HIAA/5-HT in the HIPP (Data in Brief Article Fig. 5) of the male offspring mice. No effects of perinatal PTU exposure on the monoaminergic state were observed in the frontal cortex (data not shown).

Correlations between the ambulatory activity and each index for the monoaminergic state in the five brain regions were examined using regression analysis. A significant correlation was found between the ambulatory activity and (HVA+3-MT)/DA in the ST (Fig. 5). The regression equation and coefficient of determination (R^2^) were:

SQRT(ambulation) = 33.368001 − 46.181398 × SQRT((HVA + 3-MT)/DA in the ST) (F(1, 30) = 5.3993, P = 0.0271), R^2^ = 0.152527

where SQRT indicates the square root transformation of the measurements.

Thus, the hyper-ambulatory activity was accompanied by changes in the brain monoaminergic state, and alteration of DA turnover in the ST was negatively correlated with the hyper-ambulatory activity.

### 3.5 Ambulatory response to bupropion

Hyper-ambulatory activity was accompanied by changes in the brain monoaminergic state. In particular, changes in DA turnover in the ST may be involved in the hyper-ambulatory activity. BUP inhibits the DA transporter, increases extracellular DA levels in the ST, and thus, promotes ambulation in mice [37]. Therefore, the ambulatory response to BUP was examined to test whether changes in the dopaminergic state in the ST underlie the hyper-ambulatory activity.

**Fig. 6:**
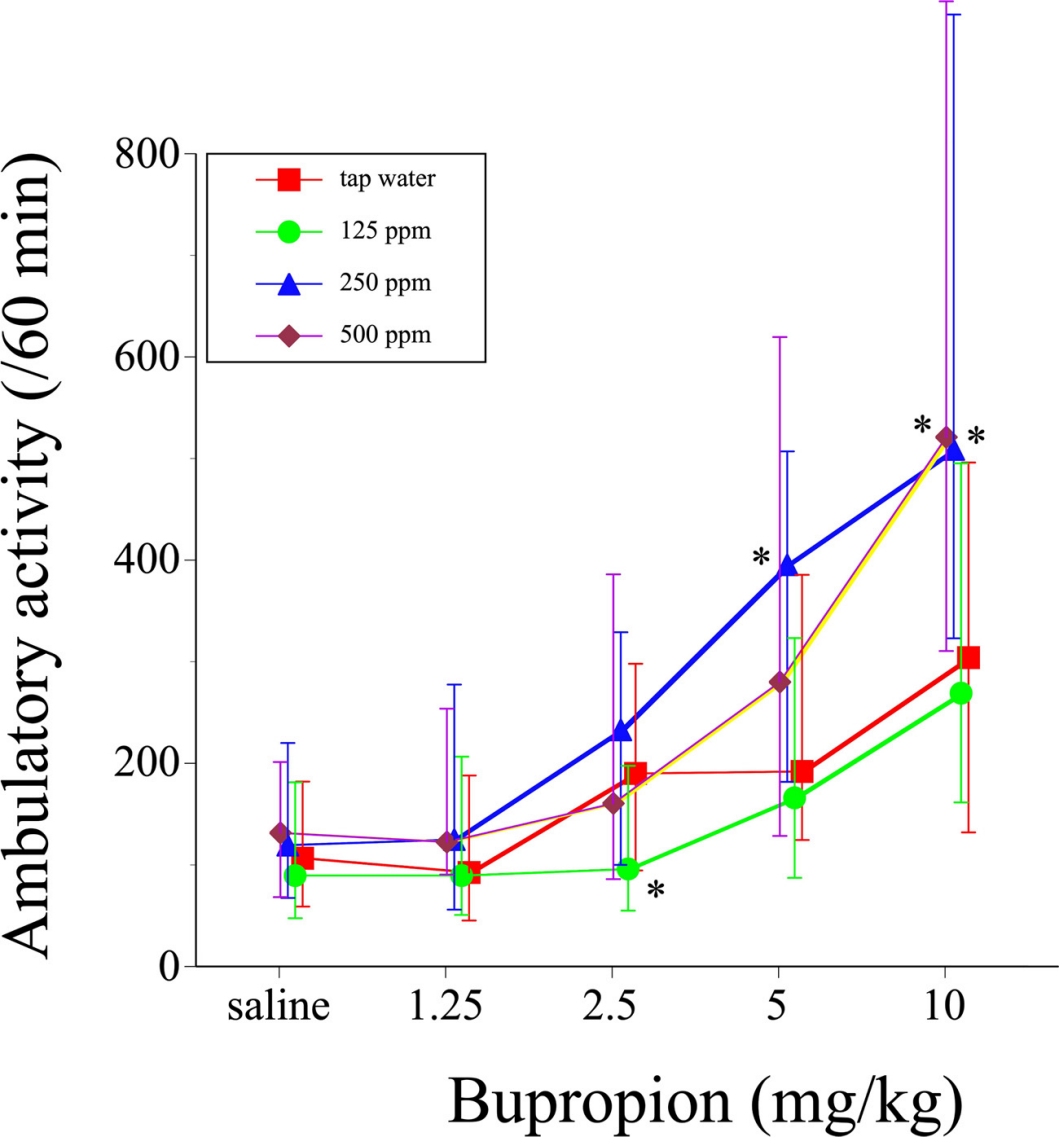
Dose-response relationships for the ambulation-promoting effect of bupropion (BUP) in male offspring mice given tap water or 125, 250, or 500 ppm PTU solution from GD15 to PND25. Symbols show median values of the ambulatory activity for 60 min after administration of saline or 1.25, 2.5, 5, or 10 mg/kg BUP. Vertical lines denote the first and third quartiles. *P < 0.05 compared with the saline control, assessed with the Wilcoxon test. N = 36–46 animals per group.

Different male offspring mice given tap water or 125–500 ppm PTU solution from GD15 to PND25 were prepared, and were used. After 30 min adaptation period, saline or 1.25, 2.5, 5, or 10 mg/kg BUP was subcutaneously administered, followed by measurement of ambulatory activity for 60 min. The test was repeated in the same mice starting with saline, followed by BUP challenges from a low dose to a high dose at 3- to 4-day intervals. BUP produced significant ambulation-promoting effects in all treatment groups (saline; χ^2^_4_ = 33.8394, P < 0.0001, 125 ppm; χ^2^_4_ = 37.8806, P < 0.0001, 250 ppm; χ^2^_4_ = 57.5084, P < 0.0001, 500 ppm; χ^2^_4_ = 38.2188, P < 0.0001). Obtained dose-response relationships for the ambulation-promoting effect of BUP indicated that mice given 250–500 ppm PTU exhibited a significantly augmented ambulatory response to 5–10 mg/kg BUP (Fig. 6). Thus, perinatal PTU exposure augmented the ambulatory response of the mice to BUP.

## 4 Discussion

T4 was below the detectable limit in offspring mice at the end of perinatal exposure to 125–500 ppm PTU. In addition, a delay in eye opening and retardation of body weight gain were also observed in offspring mice. These results indicate that offspring mice experienced hypothyroidism following exposure of their dams to 125–500 ppm PTU from GD15 to PND25. The delay in eye opening and retardation of body weight gain were concentration dependent. On the other hand, the behavioral effects were concentration-dependent in some degree, and concentration dependency was not apparent for the PTU effects on brain monoamine contents and turnover. Concentration-dependent retardation of body weight gain was observed, although growth curves for the PTU-exposed mice were almost parallel to that for control mice after termination of PTU exposure. This could be due to rapid elimination of PTU and recovery of TH levels after termination of PTU exposure [45]. Such rapid recovery of TH levels may be involved in the concentration-response relationships for the PTU effects on behaviors and brain monoamine contents in the grown-up mice, in addition to individual differences.

Ambulatory activity was measured using the tilting cage method, and therefore, ambulatory activity represents motor activity of mice in the cage. The tilting cage method is more sensitive to horizontal movement such as locomotion of the mouse in the activity cage than to vertical movement [37]. The response rate in the two-way active avoidance test is the average number of interruptions of two sets of photo beam sensors horizontally placed in the experimental chamber. Therefore, the response rate represents the degree of horizontal movement of the mouse in the experimental chamber. Psychoactive agents that increase ambulatory activity increase the response rate in the avoidance test [19,35,36], suggesting that common mechanisms underlie both ambulatory activity and the response rate in the avoidance test.

In the present study, we observed hyper-ambulatory activity and an increased response rate in the avoidance test in offspring mice with perinatal hypothyroidism. The hyperactive characteristics were evident in male offspring, but not in female offspring. TH produces its physiological effects through TRα, TRβ, and indirect molecular pathways, and the roles of TRα and TRβ for locomotor activity are likely different in mice. Mice expressing human mutant TRβ1 and TRβ-KO mice exhibit hyper-locomotor activity [27,33], whereas TRα-KO mice do not exhibit hyper-locomotor activity [27,40]. These mutant mice lack TRs during their entire life. The current study revealed that hypothyroidism that was limited to the perinatal period produced hyperactive behavioral phenotypes in mice in a male-specific manner.

DA and 5-HT systems in mouse brain considerably develop during the perinatal period [26]. TRs are widely distributed in brain and start to express at GD14, rapidly increase after birth and reach a peak by PND6 in the fetal rat brain [2,29]. TH regulates gene expression to control　 various cellular processes during brain development such as the neuronal growth and synaptogenesis through nuclear TRs and indirect molecular pathways [28], and therefore, abnormal TH state can disturb the cellular processes of brain development [1]. TRα-KO mice show significantly lower DA turnover in the ST and the dorsal raphe nucleus, but normal levels of 5-HT turnover in many brain regions [27]. In contrast, TRβ-KO mice show significantly higher DA contents and lower DA turnover in the ST and the NAcb, and significantly lower 5-HT turnover in many brain regions [27]. Thus, TRα and TRβ play different roles in brain monoaminergic systems. However, little was known about the effects of perinatal hypothyroidism on brain monoaminergic systems. In the present study, we observed significant decreases in DA turnover in the ST, NAcb, and HYPO, and a significant decrease in 5-HT turnover in the ST, NAcb, HYPO, and HIPP in mice with perinatal hypothyroidism. The findings suggest that hypothyroidism limited to the perinatal period influences development of brain monoaminergic systems to produce the low DA and 5-HT turnover in various brain regions in offspring mice.

Ambulatory and/or locomotor activity is related to DA tone in the ST [12,37]. We observed a significant correlation between ambulatory activity and DA turnover in the ST in mice with perinatal hypothyroidism. In addition, we observed that mice with perinatal hypothyroidism exhibited an augmented ambulatory response to BUP, which increases extracellular DA levels in the ST to promote mouse ambulation [37]. These observations suggest that alteration in the DA tone in the ST is involved in hyper-ambulatory activity in mice with perinatal hypothyroidism. The response rate in the avoidance test represents the degree of horizontal movement of the mouse in the experimental chamber. Psychoactive agents that promote mouse ambulation and/or locomotion through DA in the ST, such as BUP [37], amphetamine, and methamphetamine [16,20], increase the response rate in the avoidance test [19,35], indicating that DA in the ST underlies both ambulatory activity and the response rate in the avoidance test. Thus, alteration in the DA tone in the ST may also be involved in the increased response rate in the avoidance test in mice with perinatal hypothyroidism.

The hyperactive behavioral phenotypes of mice with perinatal hypothyroidism were male specific. This phenomenon is of interest, as the prevalence of ADHD is higher in boys than in girls in the ratio of 3–4:1 [7]. Testosterone influences the nigrostriatal DA system [31,34] and motor activity through the DA system [18]. Therefore, the effects of testosterone on the DA system may underlie the male-specific hyperactive behavioral phenotypes in mice with perinatal hypothyroidism. Alternatively, perinatal hypothyroidism may influence the development of brain monoaminergic systems differently in male and female mice through interactions among TRs and sex hormone receptors. Interactions of TRs and sex hormone receptors in brain cells through both nongenomic and genomic mechanisms are known [8,10,43].

A significant decrease in 5-HT turnover was also observed in the ST in mice with perinatal hypothyroidism. As mouse locomotion involves interactions between DA and 5-HT [3,13,30,38], alterations in not only the DA state but also the 5-HT state in the ST may be involved in the hyper-ambulatory activity in mice with perinatal hypothyroidism. In addition, significant decreases in DA and 5-HT turnover were observed in not only the ST but in other brain regions such as the NAcb, HYPO, and HIPP in the mice. The mice likely possessed other behavioral characteristics in addition to the hyperactive behavioral phenotypes due to the altered DA and 5-HT state in the NAcb, HYPO, and HIPP. Elucidating all behavioral phenotypes in mice with perinatal hypothyroidism requires future research.

In conclusion, the present study revealed the hyperactive behavioral phenotypes and the altered monoaminergic state in various brain regions in male mice with perinatal hypothyroidism. Alteration in DA tone in the ST was likely involved in the hyperactive behavioral phenotypes.

## Declaration of Competing Interest

The authors declare that they have no known competing financial interests or personal relationships that could have appeared to influence the work reported in this paper.
